# Adipose Tissue Lactate Clearance but Not Blood Lactate Clearance Is Associated with Clinical Outcome in Sepsis or Septic Shock during the Post-Resuscitation Period

**DOI:** 10.3390/metabo8020028

**Published:** 2018-04-21

**Authors:** Ioannis Ilias, Sofia Apollonatou, Dimitra-Argyro Vassiliadi, Nikitas Nikitas, Maria Theodorakopoulou, Argyris Diamantakis, Anastasia Kotanidou, Ioanna Dimopoulou

**Affiliations:** 1 Endocrine Unit, Elena Venizelou Hospital, Athens 11521, Greece; 2 Second Department of Critical Care Medicine, Attikon University Hospital, National and Kapodistrian University of Athens, Medical School, Athens 10462, Greece; sofapol@yahoo.gr (S.A.); Nikitas.nikitas@outlook.com (N.N.); Mariatheodor10@gmail.com (M.T.); argyris-d@hotmail.com (A.D.); 3 Department of Endocrinology, Evangelismos Hospital, Athens 10676, Greece; Dimitra.vas@googlemail.com; 4 First Department of Critical Care Medicine, Evangelismos Hospital, National and Kapodistrian University of Athens, Medical School, Athens 10676, Greece; akotanid@med.uoa.gr (A.K.); idimo@otenet.gr (I.D.)

**Keywords:** microdialysis, intensive care unit, sepsis, lactate clearance, tissue hypoxia, outcome

## Abstract

No study has directly measured tissue lactate clearance in patients with sepsis during the post-resuscitation period. In this study we aimed to assess in ICU patients with sepsis (*n* = 32) or septic shock (*n* = 79)—during the post-resuscitation phase—the relative kinetics of blood/tissue lactate clearances and to examine whether these are associated with outcome. We measured serially—over a 48-h period—blood and adipose tissue interstitial fluid lactate levels (with microdialysis) and we calculated lactate clearance. Statistics included mixed model analysis, Friedman’s analysis of variance, Wilcoxon’s test, Mann-Whitney’s test, receiver operating characteristics curves and logistic regression. Forty patients died (28-day mortality rate = 28%). Tissue lactate clearance was higher compared to blood lactate clearance at 0–8, 0–12, 0–16, 0–20 and 0–24 h (all *p* < 0.05). Tissue lactate clearance was higher in survivors compared to non-survivors at 0–12, 0–20 and 0–24 h (all *p* = 0.02). APACHE II along with tissue lactate clearance <30% at 0–12, 0–20 and 0–24 h were independent outcome predictors. We did not find blood lactate clearance to be related to survival. Thus, in critically ill septic patients, elevated tissue (but not blood) lactate clearance, was associated with a favorable clinical outcome.

## 1. Introduction

Sepsis refers to the infectious syndrome complicated by acute organ dysfunction, whereas septic shock represents the most severe clinical situation of sepsis, characterized by hypotension not reversed with fluid resuscitation. The end-result is inadequate perfusion, tissue hypoxia and organ failure, as a consequence of oxygen supply-demand imbalance at the cellular level [1,2].

Traditionally, blood lactate, an easily measured metabolite, is used for the assessment of tissue perfusion and oxidative status. According to current guidelines, blood lactate is the only biomarker recommended for diagnosis and stratification of sepsis and septic shock [3]. Initial lactate levels, serial lactate concentrations, the area under the curve of increased lactate levels and lactate clearance have all been well correlated to morbidity and mortality [4,5,6,7,8,9,10,11]. While it is widely accepted that blood lactate and its clearance are linked to survival, it remains currently disputed whether these should be used as an end-point of early goal-directed therapy in critical ill septic patients [3]. This is largely based on the knowledge that in critically patients other processes unrelated to tissue hypoxia may increase lactate levels; these include increased glycolysis, mitochondrial dysfunction, defects in pyruvate metabolism or prolonged lactate clearance due to liver dysfunction [11].

According to current critical care management guidelines, the overall hemodynamic supportive therapy (resuscitation phase) seeks to restore macrocirculatory oxygenation [3]. Microcirculatory perfusion could be inadequate, and if prolonged it may be associated with worse prognosis. Such findings have driven new technologies that can assess microcirculation in situ [12,13]. The cells are surrounded by interstitial fluid, the composition of which reflects their metabolic activity, and hence restoration of tissue perfusion has been targeted [14]. Indeed, monitoring of the interstitium is feasible with the implementation of minimally invasive techniques, including microdialysis (MD) [15,16,17,18]. MD is now increasingly used in critically ill septic patients as a research tool and the measurement of metabolites, such as lactate, pyruvate and glycerol together with the calculation of the lactate/pyruvate (L/P) ratio, directly assess energy metabolic disorders at the tissue level [18,19,20,21,22,23,24,25,26,27,28,29,30]. In this regard, a recent study by our group examined the interplay between adipose tissue and blood lactate and found that in ICU patients with septic or cardiogenic shock the rise in tissue lactate preceded the increase in blood lactate [19].

In the present study, and to further expand our knowledge on lactate kinetics in critically ill septic patients, we measured lactate clearance (i.e., the percent decrease in lactate) in blood and adipose tissue in ICU patients with sepsis or septic shock over a 48-h period, during the post-resuscitative period. The purpose was first, to examine the potential differences in lactate clearances between blood and extracellular fluid and second, to investigate whether these are associated with clinical outcome. Extracellular fluid was obtained after inserting a MD catheter into the subcutaneous adipose tissue of the upper thigh.

## 2. Results

### 2.1. Patients’ Characteristics

During the study period 190 critically ill patients underwent investigation with MD. Of the 190 patients, 100 patients had septic shock and 40 patients had sepsis (total 140 patients). Of the 140 patients, 111 were included in the current investigation, having either sepsis (*n* = 32) or septic shock (*n* = 79). Twenty-nine patients with septic shock or sepsis were excluded, either due to death within the first 48-h after ICU admission (*n* = 9) or due to missing values (lack of samples) related to the MD technique (*n* = 20). Table 1 shows their main demographic and clinical characteristics on admission in the ICU.

The most common infection was pneumonia followed by intra-abdominal infection. Total bilirubin was used as an index of liver dysfunction, as a part of the SOFA score. Median bilirubin was 0.70 mg/dL (range: 0.20 to 20.50 mg/dL). Nineteen patients had liver damage, defined as bilirubin levels > 1.90 mg/dL.

Forty patients died, yielding a 28 day-mortality rate of 36%. Non-survivors had higher median APACHE II (22 vs. 18, *p* < 0.001) and SOFA (9 vs. 7, *p* = 0.04) scores, while age was similar in the two groups (71 vs. 65 years, *p* = 0.18). There were no differences in CRP between survivors and non-survivors (median: 130 vs. 85 mg/L, *p* = 0.11).

### 2.2. Blood/Adipose Tissue Lactate Concentrations and Clearances in the Entire Cohort

**Figure 1:**
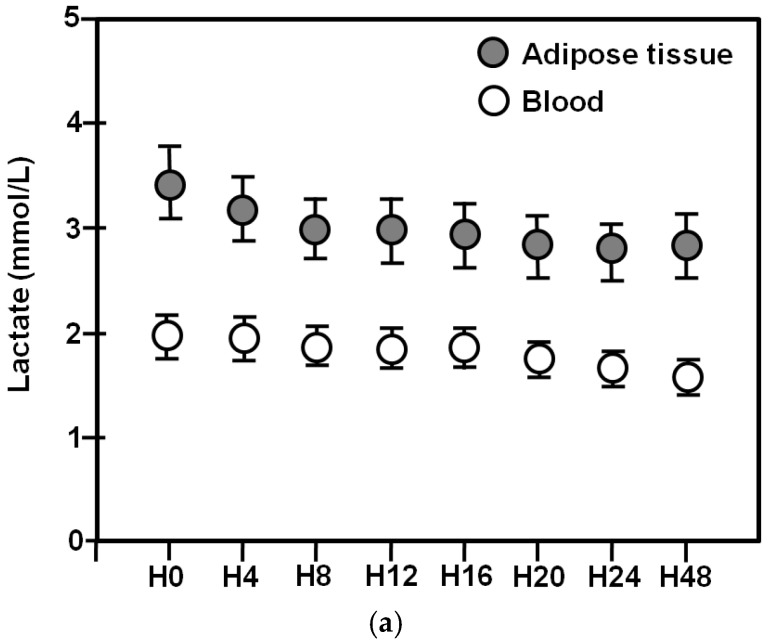

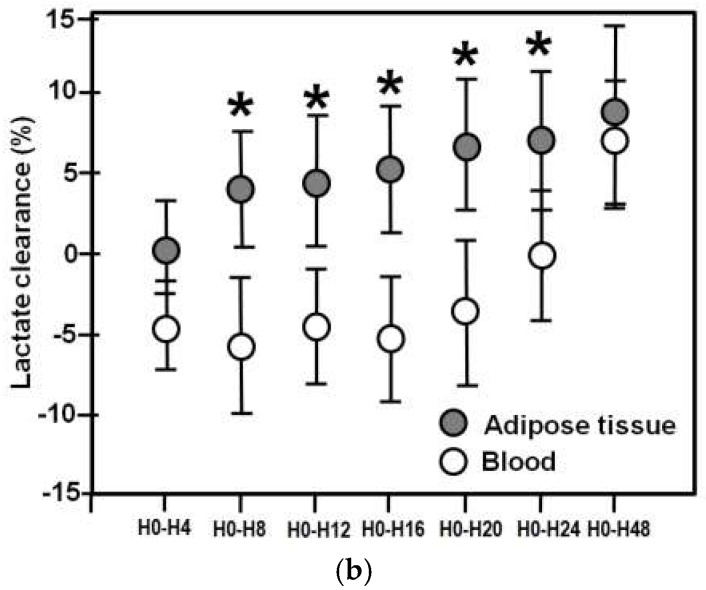
(**a**) Mean (± SE) lactate levels in adipose tissue (closed circles) and in blood (open circles) over 48 h; (**b**) Mean (± SE) lactate clearances in adipose tissue (closed circles) and in blood (open circles) over 48 h. Asterisks denote statistically significant differences between them.

Adipose tissue and blood lactate levels for the entire 48-h period are shown in Figure 1a.

Mixed model analysis revealed that adipose tissue lactate dropped significantly over time (*p* < 0.001). In particular, H0 was higher compared to H12, H16, H20, H24, and H48. Similarly, blood lactate also decreased (*p* < 0.001). More precisely, H0 was higher compared to H48. In contrast, there were no differences in blood lactate levels between H0 and H4, H8, H12, H16, and H24. At all time points adipose tissue lactate was significantly higher than blood lactate (*p* < 0.001) (Wilcoxon rank-sum test).

Adipose tissue and blood lactate clearances are shown in Figure 1b.

Friedman analysis of variance by ranks showed that blood and tissue lactate clearances increased significantly over time (*p* < 0.001, for both). Tissue lactate clearance was significantly higher compared to blood lactate clearance at H0–H8 (*p* = 0.02), H0–H12 (*p* = 0.008), H0–H16 (*p* = 0.01), H0–H20 (*p* = 0.01), and H0–H24 (*p* = 0.02) (Wilcoxon rank-sum test). Of note, mean values in adipose tissue lactate clearance were positive throughout the 48 h observation period, whereas, blood lactate clearance was negative during the whole 24 h period. The first positive blood lactate clearance was obtained at 48 h.

### 2.3. Blood/Adipose Tissue Lactate Clearances in Survivors and Non-Survivors

**Figure 2:**
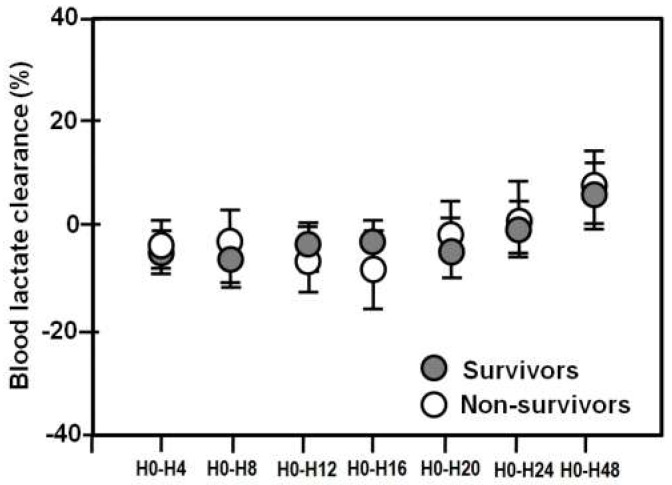
Mean (± SE) blood lactate clearances in survivors (closed circles) and non-survivors (open circles) over 48 h.

Friedman’s analysis of variance by ranks showed that blood and adipose tissue lactate clearance increased both in survivors (*p* = 0.001) and in non-survivors (*p* = 0.03). There were no significant differences in blood lactate clearances between the two groups (*p* values 0.7–0.9) (Mann-Whitney rank sum test) (Figure 2).

**Figure 3:**
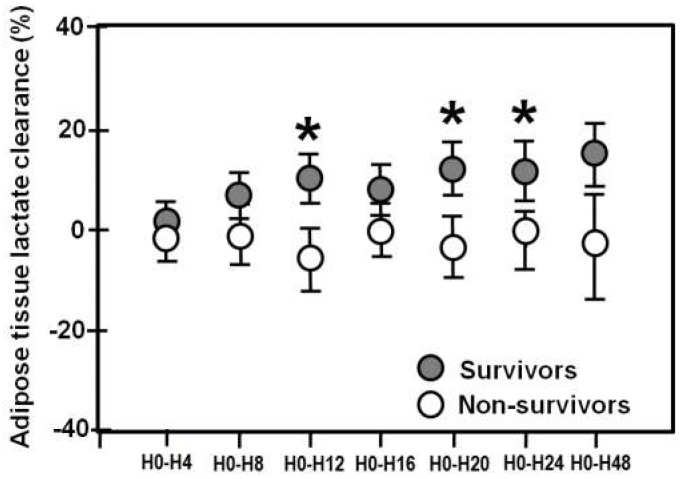
Mean (± SE) adipose tissue lactate clearances in survivors (closed circles) and non-survivors (open circles) over 48 h. Asterisks denote statistically significant differences between the two groups.

Adipose tissue lactate clearance increased significantly over time in survivors (*p* < 0.001), and remained unchanged in non-survivors (*p* = 0.09). Tissue clearance was higher in survivors compared to non-survivors at H0–H12 (*p* = 0.02), H0–H20 (*p* = 0.02), and H0–H24 (*p* = 0.02) (Figure 3).

ROC curves for the overall ability of lactate adipose tissue clearance, at H0–H12, H0–H20, and H0–H24, to discriminate between survivors and non-survivors were constructed. We found that a lactate adipose tissue clearance below 30%, identified non-survivors from survivors, with sensitivities of 93%, 83% and 80%, and specificities of 27%, 40% and 43%, at H0–H12, H0–H20, and H0–H24, respectively. Multivariate logistic regression analysis, which included the APACHE II score (OR = 1.2, CI = 1.1–1.3, *p* < 0.001), showed that the 30% cutoff value in tissue lactate clearance was an independent outcome predictor (OR = 4.2, 95% CI = 1.1–16.0, *p* = 0.03 for tissue lactate clearance at H0–H12; OR = 3.2, CI = 1.2–8.6, *p* = 0.02, for tissue lactate clearance at H0–H20; OR = 3.0, CI = 1.2–7.7, *p* = 0.03, for tissue lactate clearance at H0–H12).

## 3. Discussion

This investigation indicates that in critically ill patients with sepsis or septic shock during the post-resuscitative phase, lactate clearance in adipose tissue was positive, and followed a progressive, incremental pattern. In contrast, blood lactate clearance showed a biphasic pattern, i.e., it was negative initially and then with a delay of 24 h, exhibited an incremental pattern. Importantly, high adipose tissue lactate clearance, but not high blood lactate clearance, was associated with better survival rates. Multivariate analysis showed that lactate adipose tissue clearances at H0–H12, H0–H20 or H0–H24 < 30% along with clinical severity scores, were independent outcome predictors. To our knowledge, this is the first study to investigate the relative kinetics in blood and tissue lactate clearances and to describe an association between tissue lactate clearance and clinical outcome in ICU septic patients.

Lactic acid may be considered as the endpoint of the anaerobic glucolysis. In the liver periportal hepatocytes clear almost 70% of lactate and by the metabolic processes of gluconeogenesis and, to a lesser extent, oxidation. Mitochondria-enriched tissues, such as skeletal and cardiac muscle cells and renal proximal tubule cells remove the remaining circulating lactate by converting it to pyruvate. Ultimately, less than 5% of lactate is renally excreted [11,31].

The prognostic value of blood lactate clearance in critically ill patients has already been recognized in earlier studies. In 1993 Abramson D. et al. reported that lactate clearance, defined as a decrease of lactate to less than 2 mmol/L by 24 h, was a predictor of survival in multiple trauma patients [4]. Since then, a number of studies have tested the outcome predictive ability in diverse populations of critically ill patients, and have confirmed that early normalization (within the first 6 h) of lactate levels are associated with decreased mortality rates [6,7,8,9,10]. Among these, an important study in this field is the one conducted by Nguyen and coworkers and deserves special consideration. The authors included 111 patients with sepsis and septic shock and measured serially blood lactate levels. The fist sample was obtained before ICU admission in the emergency department and thereafter at hour 6, and over the first 72 h of hospitalization. Increased lactate clearance during the initial resuscitation phase of sepsis management was associated with improved mortality rates. They concluded, that early lactate clearance may reflect resolution of global tissue hypoxia [7].

In our investigation, patients were studied during the post-resuscitative phase; blood lactate clearance at study entry (i.e., H0–H4) was negative, having a mean value of −4.4 ± 29.3%, and preserved negative values during the first 24 h. The first positive value (6.8 ± 41.7%) was obtained at the time period H0–H48. Blood lactate clearance increased both in survivors and non-survivors over time, however, there were no differences between the two groups. Such results are quite contradictory compared to those reported by others [4,6,7,8,9,10]. This might be related to the fact that most previous studies on the prognostic ability of blood lactate clearance have been carried out during the very early period of sepsis onset (in the first 6 h), and more importantly, lactate clearance has been used as a marker of effective resuscitation in early septic shock. For instance, in the classical study of Nguyen et al., blood lactate clearance at H0–H6 was +27.1 ± 44.4%, where H0 is presentation at the emergency department and H6 six hours later, and of importance, following the implementation of intense therapeutic interventions [7]. In contrast, our patients were studied during the sub-acute phase, while being clinically stable, essentially during the post-resuscitation period. Furthermore, in the present investigation, and in contrast to others, we did not use lactate clearance as an end-point of hemodynamic resuscitation or of any other intense treatment.

Currently, minimally invasive techniques that monitor locally targeted organs or tissues are available, in particular MD. This method enables monitoring of energy-related metabolites within the interstitial space, including lactate levels. In particular, tissue lactate measurement in severely ill patients has gained increased attention and has provided valuable pathophysiological information [25,26]. Levy et al. showed that in septic shock skeletal muscles generate lactate [27] and that in sepsis the muscle-to-serum lactate gradient is predictive of progression to septic shock [28]. Our group found that adipose tissue-derived lactate is higher in septic shock compared to sepsis, indicating the presence of an association between tissue lactate and sepsis severity [20]. Subsequently, we demonstrated that in septic shock, adipose tissue L/P ratio is independently associated with clinical outcome, carrying a predictive capability similar to that of APACHE II score [24]. In a very recent study, we found that in patients suffering from shock (septic or cardiogenic), the rise in tissue lactate preceded the increase in blood lactate by 4 h, suggesting that MD may detect subtle metabolic events before these are evident in the systemic circulation [21].

To further explore lactate kinetics, in the current study we focused on adipose tissue lactate clearance in stable ICU patients with sepsis or septic shock. In our patients, and contrasting blood lactate clearance, tissue lactate clearance was positive starting from H0–H4, remained positive throughout, and increased significantly during the entire 48-h observation period. It has been previously shown that in experimental human sepsis lactate production does not occur in the muscles [32], and our data raise the possibility that site of lactate generation in sepsis might be the subcutaneous adipose tissue. Moreover, adipose tissue lactate clearance was found to be significantly higher in survivors compared to non-survivors. Finally, tissue lactate clearance of below 30% constituted an independent outcome predictor together with clinical severity scores. As discussed before, lactate is measured in blood as a surrogate for the magnitude of tissue hypoperfusion. Blood endpoints, however, may not always reveal the changes in the cell milieu [14]. Yet most biochemical events take place in tissues, and thus tissue lactate might be a better marker for assessing tissue oxygenation than blood lactate. The present data for the first time showed that lactate elimination in tissue precedes the lactate fall in blood and this pattern is associated with higher survival rates. These in turn indicate that MD may detect metabolic events that would go undetected by conventional sampling approaches, as described in neurointensive care [15,16,17]. Furthermore, the present study supports the concept of significant discrepancies between plasma and interstitial compound concentrations in severely ill patients. For instance, it is known that anti-microbial unbound concentrations at the tissue may be sub-inhibitory, although effective concentrations are achieved in serum [29]. Only the unbound concentration in the interstitium, however, is considered to contribute to the efficacy of anti-infectives. Similarly, a moderate correlation or a lack or correlation has been observed between serum and tissue cortisol levels in sepsis or burn injury [23,30]. From a pathophysiological point of view the equilibrium between plasma and tissue interstitial space in the setting of sepsis may be incomplete due to interstitial edema, impairment of the microcirculation or administration of vasopressor agents [14].

Our study has some limitations that need to be considered. It is a single-center study with a selected sample of critically ill septic patients and, therefore, its findings may not apply to other settings. No other endpoints of lipid metabolism such as glycerol or free fatty acids (FFAs) were assessed with MD (the latter due to their high molecular weight could not pass through the MD catheter used). In the present study the MD catheter was inserted in the subcutaneous adipose tissue. It would be of interest to see whether similar results can be obtained if the catheter is placed in other metabolically active tissues, for instance in peripheral muscles. Local evaluation of tissue perfusion is helpful and more accurate than global/systemic evaluation, however no other tissue perfusion parameters such as mottling or urinary output or sublingual capillaroscopy were implemented. Furthermore, arterial lactate levels were not very high in the patients studied (thus being incongruous with previous studies) and after the early resuscitation period, lactate clearance was very low, around 5%. Despite these limitations, we contend that observational studies are useful in generating hypotheses and guiding further study design.

## 4. Materials and Methods

### 4.1. Subjects

This prospective study included all critically ill patients with sepsis or septic shock admitted to the 25-bed medical-surgical adult ICU of “Attikon” University Hospital between March 2008 and July 2012 and treated using a standardized resuscitation protocol. Approval for the present study was given by the “Attikon” University Hospital’s Institutional Review Board/Ethics Committee. The study’s approval code/number was 478, given on 2 February 2011. Some of the patients in this study were included in previously published research from our group. Sepsis and septic shock were defined according to The Third International Consensus Definitions for Sepsis and Septic Shock task force criteria [33]. Some of the patients in this study were included in previously published research from our group [19,20,21,22,23,24]. Patients were excluded from the study if they were younger than 18 years old, if they were mechanically ventilated for more than 48 h before ICU admission or had no need for intubation and mechanical ventilation during ICU stay (mechanical ventilation may lower blood lactate [34]). Further exclusion criteria included do-not-resuscitate clinical conditions, diagnosis of brain-death upon ICU entry and presence of HIV infection (that increases blood lactate [35]).

### 4.2. Microdialysis

As previously described [15,16,17,19,20], a MD catheter (CMA 60, CMA Microdialysis AB, Stockholm, Sweden) was inserted, under sterile conditions, into the subcutaneous adipose tissue of the upper thigh of the study’s patients. The catheter was inserted within 24 h from ICU admission and between 04:00 and 08:00 h (Day 1). The data’s zero point (Ho) was considered at two hours after the MD probe was inserted and set to stably run. On day 1, and for the following 24 h 72 μL of MD fluid samples were collected in 4-h intervals, starting from 5:00 a.m. Lactate concentration was determined in each microdialysate sample. The 4-h sample collection intervals throughout the day were the following: (1) 05:00–09:00; (2) 09:00–13:00; (3) 13:00–17:00; (4) 17:00–21:00; (5) 21:00–01: 00; (6) 01:00–05:00. The last MD sample was collected within 48 h of ICU admission. An automated analyzer (single-beam filter photometer; CMA 600 Microdialysis Analyzer, CMA Microdialysis AB, Stockholm, Sweden; imprecision for any dialysate—including interstitial fluid—was provided by the manufacturer as being ≤6% CV [36]) was used for the determination of tissue lactate concentration.

### 4.3. Other Measurements

Daily blood sampling was done for routine hematological and biochemistry tests. Concomitantly with MD sampling, arterial blood samples were obtained for lactate measurement (GEM Premier 3000, Instrumentation Laboratory, Milan, Italy).

### 4.4. Blood and Tissue Lactate Clearance Definitions

Blood and tissue lactate clearance was defined by the equation [lactate_initial_ − lactate_delayed_/lactate_initial_] × 100%. Lactate_initial_ is blood or tissue lactate within 24 h after admission to the ICU (H0). Lactate_delayed_ is blood or tissue lactate concentration after 4, 8, 12, 16, 20, 24 and 48 h, (denoted as H4, H8, H12, H16, H20, H24, and H48, respectively). A positive value indicates a decrease in lactate, while a negative value denotes an increase in lactate levels [7,10].

### 4.5. Data Collection

In all patients the following variables were recorded: age, gender, site of infection, and sepsis stage (sepsis or septic shock). The Acute Physiology and Chronic Health Evaluation (APACHE II) and the Sequential Organ Failure Assessment (SOFA) scores were calculated on day 1 [37,38]. Finally, ICU outcome (28-day mortality rate) was recorded.

### 4.6. Statistical Analysis

All data were tested for normal distribution by the Kolmogorov-Smirnov test. Results are presented as means ± SD, medians and ranges or counts. Mixed model analysis was carried out to assess the evolution of blood and adipose tissue lactate in the entire cohort over time. Friedman analysis of variance by ranks was used to assess: (i) the evolution of blood and tissue lactate clearance over 48-h; (ii) the evolution in blood and tissue lactate clearance in survivors and non-survivors during the entire observation period. The Wilcoxon rank-sum test was applied to compare: (i) adipose tissue and blood lactate levels at the various time points (H0, H4, H8, H12, H16, H20, H24, and H48); (ii) blood and adipose tissue lactate clearances at various time periods (H0–H4, H0–H8, H0–H12, H0–H16, H0–H20, H0–H24, and H0–H48). Mann-Whitney rank sum test was used to compare: (i) blood and tissue lactate clearances in survivors and non-survivors at the various time points (H0–H4, H0–H8, H0–H12, H0–H16, H0–H20, H0–H24, and H0–H48); (ii) survivors and non-survivors with regard demographic data, and clinical severity scores. Receiver operating characteristics (ROC) curves were constructed to find the best cut-off value with regard to adipose tissue lactate clearance at H0–H12, H0–H20, and H0–H24 and mortality prediction. Variables yielding *p* < 0.05 in the univariate analyses were entered into multiple logistic regression models. The selection of the variables was made with the backward elimination method. Odds ratios (OR) and 95% confidence intervals (CI) are reported. In all analyses a *p* < 0.05 was considered statistically significant.

## 5. Conclusions

Adipose tissue MD appears to be a suitable means of obtaining important information about cellular metabolic changes in adipose tissue of critically ill septic patient, and can detect early changes that laboratory parameters can identify only later and incompletely. Outcome improvements have been well-established following the use of MD in the setting of brain injury where this technique is widely implemented [17]. Future studies would be valuable to define the place of MD in the daily monitoring and management of critically ill septic patients.

## Figures and Tables

**Table 1:** Baseline demographic, clinical, and laboratory parameters of the study population (*n* = 111) *.

Age (years)	67 (24–92)
Gender (men/women)	71/40
APACHE II score	19 (5–32)
SOFA score	8 (2–15)
Mechanical ventilation	111
HR (beat/min)	94 (52–200)
MAP (mmHg)	77 (71–120)
Temperature (°C)	36.5 (35.0–39.6)
White blood cell count (×10^3^/μL)	12.400 (12.100–47.910)
Hemoglobin (g/L)	9.9 (5.7–16.4)
Creatinine (mg/dL)	1.6 (0.3–7.9)
Bilirubin (mg/dL)	0.70 (0.20–20.50)
PO_2_/FIO_2_	203 (71–440)
C-reactive protein (mg/L)	130 (71–470)

* Values are expressed as medians (and ranges) or counts; **Abbreviations**: APACHE, Acute Physiology and Chronic Health Evaluation; SOFA, Sequential Organ Failure Assessment; HR, heart rate; MAP, mean arterial pressure; PO_2_/FIO_2_, ratio of the partial pressure of arterial O_2_ to the fraction of inspired O_2_.
